# Supplementary material for: Unveiling the Bioactive Potential of the Invasive Jellyfish Phyllorhiza punctata Through Integrative Transcriptomic and Proteomic Analyses
Source: Biomolecules. 2025 Aug 4;15(8):1121. doi: 10.3390/biom15081121 (PMC12383608; doi:10.3390/biom15081121)
Supplement: Supplementary file 1 [file biomolecules-15-01121-s001.zip › Suplementary Material/FileS3/PEPFOLD4/XP_065071994_7820/clusters.html]

 PEP-FOLD clusters


Download archive of all models  here   

|  |  |  |
| --- | --- | --- |
| # | file | sOPEP |
| # cluster 1 - Size: 68 |  |  |
|  | XP\_065071994\_7820-model1 | -11.2082 |
|  | XP\_065071994\_7820-model1.1 | -10.9774 |
|  | XP\_065071994\_7820-model1.2 | -10.9774 |
|  | XP\_065071994\_7820-model1.3 | -10.9774 |
|  | XP\_065071994\_7820-model1.4 | -10.9774 |
|  | XP\_065071994\_7820-model1.5 | -10.9491 |
|  | XP\_065071994\_7820-model1.6 | -10.9491 |
|  | XP\_065071994\_7820-model1.7 | -10.9491 |
|  | XP\_065071994\_7820-model1.8 | -10.9491 |
|  | XP\_065071994\_7820-model1.9 | -10.9491 |
|  | XP\_065071994\_7820-model1.10 | -10.9491 |
|  | XP\_065071994\_7820-model1.11 | -10.9491 |
|  | XP\_065071994\_7820-model1.12 | -10.9491 |
|  | XP\_065071994\_7820-model1.13 | -10.9491 |
|  | XP\_065071994\_7820-model1.14 | -10.9491 |
|  | XP\_065071994\_7820-model1.15 | -10.9491 |
|  | XP\_065071994\_7820-model1.16 | -10.9491 |
|  | XP\_065071994\_7820-model1.17 | -10.9491 |
|  | XP\_065071994\_7820-model1.18 | -10.9491 |
|  | XP\_065071994\_7820-model1.19 | -10.9491 |
|  | XP\_065071994\_7820-model1.20 | -10.9491 |
|  | XP\_065071994\_7820-model1.21 | -10.9491 |
|  | XP\_065071994\_7820-model1.22 | -10.9491 |
|  | XP\_065071994\_7820-model1.23 | -10.9491 |
|  | XP\_065071994\_7820-model1.24 | -10.9491 |
|  | XP\_065071994\_7820-model1.25 | -10.9491 |
|  | XP\_065071994\_7820-model1.26 | -10.9491 |
|  | XP\_065071994\_7820-model1.27 | -10.9491 |
|  | XP\_065071994\_7820-model1.28 | -10.9491 |
|  | XP\_065071994\_7820-model1.29 | -10.9491 |
|  | XP\_065071994\_7820-model1.30 | -10.9491 |
|  | XP\_065071994\_7820-model1.31 | -10.9491 |
|  | XP\_065071994\_7820-model1.32 | -10.9491 |
|  | XP\_065071994\_7820-model1.33 | -10.9491 |
|  | XP\_065071994\_7820-model1.34 | -10.9491 |
|  | XP\_065071994\_7820-model1.35 | -10.9491 |
|  | XP\_065071994\_7820-model1.36 | -10.9491 |
|  | XP\_065071994\_7820-model1.37 | -10.9491 |
|  | XP\_065071994\_7820-model1.38 | -10.9491 |
|  | XP\_065071994\_7820-model1.39 | -10.9491 |
|  | XP\_065071994\_7820-model1.40 | -10.9491 |
|  | XP\_065071994\_7820-model1.41 | -10.9491 |
|  | XP\_065071994\_7820-model1.42 | -10.9491 |
|  | XP\_065071994\_7820-model1.43 | -10.9491 |
|  | XP\_065071994\_7820-model1.44 | -10.9491 |
|  | XP\_065071994\_7820-model1.45 | -10.9491 |
|  | XP\_065071994\_7820-model1.46 | -10.9491 |
|  | XP\_065071994\_7820-model1.47 | -10.9491 |
|  | XP\_065071994\_7820-model1.48 | -10.9491 |
|  | XP\_065071994\_7820-model1.49 | -10.9444 |
|  | XP\_065071994\_7820-model1.50 | -10.9427 |
|  | XP\_065071994\_7820-model1.51 | -10.9427 |
|  | XP\_065071994\_7820-model1.52 | -10.9427 |
|  | XP\_065071994\_7820-model1.53 | -10.9427 |
|  | XP\_065071994\_7820-model1.54 | -10.9427 |
|  | XP\_065071994\_7820-model1.55 | -10.9427 |
|  | XP\_065071994\_7820-model1.56 | -10.9427 |
|  | XP\_065071994\_7820-model1.57 | -10.9427 |
|  | XP\_065071994\_7820-model1.58 | -10.9399 |
|  | XP\_065071994\_7820-model1.59 | -10.9399 |
|  | XP\_065071994\_7820-model1.60 | -10.9151 |
|  | XP\_065071994\_7820-model1.61 | -10.8886 |
|  | XP\_065071994\_7820-model1.62 | -10.88 |
|  | XP\_065071994\_7820-model1.63 | -10.88 |
|  | XP\_065071994\_7820-model1.64 | -10.88 |
|  | XP\_065071994\_7820-model1.65 | -10.8688 |
|  | XP\_065071994\_7820-model1.66 | -10.7905 |
|  | XP\_065071994\_7820-model1.67 | -10.1546 |
| # cluster 2 - Size: 31 |  |  |
|  | XP\_065071994\_7820-model2 | -11.0208 |
|  | XP\_065071994\_7820-model2.1 | -11.0208 |
|  | XP\_065071994\_7820-model2.2 | -11.0208 |
|  | XP\_065071994\_7820-model2.3 | -11.0208 |
|  | XP\_065071994\_7820-model2.4 | -11.0208 |
|  | XP\_065071994\_7820-model2.5 | -11.0208 |
|  | XP\_065071994\_7820-model2.6 | -11.0208 |
|  | XP\_065071994\_7820-model2.7 | -11.0208 |
|  | XP\_065071994\_7820-model2.8 | -11.0208 |
|  | XP\_065071994\_7820-model2.9 | -11.0208 |
|  | XP\_065071994\_7820-model2.10 | -11.0208 |
|  | XP\_065071994\_7820-model2.11 | -11.0208 |
|  | XP\_065071994\_7820-model2.12 | -11.0208 |
|  | XP\_065071994\_7820-model2.13 | -11.0208 |
|  | XP\_065071994\_7820-model2.14 | -11.0208 |
|  | XP\_065071994\_7820-model2.15 | -11.0208 |
|  | XP\_065071994\_7820-model2.16 | -11.0208 |
|  | XP\_065071994\_7820-model2.17 | -11.0208 |
|  | XP\_065071994\_7820-model2.18 | -11.0208 |
|  | XP\_065071994\_7820-model2.19 | -11.0192 |
|  | XP\_065071994\_7820-model2.20 | -11.0027 |
|  | XP\_065071994\_7820-model2.21 | -11.0027 |
|  | XP\_065071994\_7820-model2.22 | -11.0027 |
|  | XP\_065071994\_7820-model2.23 | -11.0027 |
|  | XP\_065071994\_7820-model2.24 | -11.0027 |
|  | XP\_065071994\_7820-model2.25 | -11.0027 |
|  | XP\_065071994\_7820-model2.26 | -10.9871 |
|  | XP\_065071994\_7820-model2.27 | -10.9871 |
|  | XP\_065071994\_7820-model2.28 | -10.9871 |
|  | XP\_065071994\_7820-model2.29 | -10.9541 |
|  | XP\_065071994\_7820-model2.30 | -10.9508 |
| # cluster 3 - Size: 1 |  |  |
|  | XP\_065071994\_7820-model3 | -9.53989 |
|  |  |  |
